# Identification of a novel *ACADSB* variant for the presymptomatic diagnosis of 2-Methylbutyryl-CoA dehydrogenase deficiency through newborn screening in Iran

**DOI:** 10.1186/s13023-025-04163-8

**Published:** 2026-01-12

**Authors:** Maryam Nasri, Nejat Mahdieh, Farzaneh Abbasi, Reihaneh Mohsenipour, Saeideh Abdolahpour

**Affiliations:** 1 Growth and Development Research Center, Children’s Medical Center, Tehran University of Medical Sciences, Tehran, Iran; 2 Cardiogenetic Research Center, Rajaie Cardiovascular Institute, Iran University of Medical Sciences, Tehran, Iran

**Keywords:** 2-Methylbutyryl-CoA dehydrogenase deficiency, Newborn screening, Organic acidemia, *ACADSB* gene, Novel variant, Genetic testing, Iran

## Abstract

**Background:**

2-Methylbutyryl-CoA dehydrogenase deficiency (2-MBDD), also known as short/branched-chain acyl-CoA dehydrogenase (SBCAD) deficiency, is a rare inborn error of metabolism classified as an organic acidemia. Early detection through neonatal screening is crucial to prevent irreversible complications. This study reports the first documented case of 2-MBDD in Iran, identified through the national neonatal screening program in 2022.

**Materials and methods:**

Metabolic screening was performed on dried blood spots (DBS) using electrospray ionization tandem mass spectrometry (ESI-MS/MS). Urine organic acid analysis was conducted via gas chromatography-mass spectrometry (GC/MS). Comprehensive clinical assessments, including ophthalmologic and audiologic evaluations, electroencephalography (EEG), echocardiography, and brain magnetic resonance imaging (MRI), were performed. Whole-exome sequencing (WES) was used to confirm the diagnosis.

**Results:**

A male neonate, delivered by cesarean section, was asymptomatic at birth. Initial metabolic screening revealed elevated 2-methylbutyrylcarnitine (C5) levels, confirmed by urine organic acid analysis and genetic testing, which identified a novel likely pathogenic variant in the *ACADSB* gene (c.907G > C; p.G303R). The infant was managed with a carnitine-supplemented diet and continued breastfeeding. Regular follow-ups demonstrated normal growth, neurodevelopmental milestones, and biochemical parameters, with no abnormalities detected. Post-treatment, C5 levels stabilized at 0.4 µmol/L, within the intermediate range.

**Conclusion:**

This case underscores the pivotal role of neonatal screening in the early diagnosis and management of rare metabolic disorders. Timely intervention can prevent severe complications and improve clinical outcomes, highlighting the need for expanded newborn screening programs and population-specific genetic studies.

## Background

Inborn errors of metabolism (IEMs) are a diverse group of rare genetic disorders caused by enzymatic or transporter defects in metabolic pathways. These defects lead to the accumulation of toxic metabolites, resulting in clinical complications, such as metabolic acidosis, growth delays, and neurological impairments [1]. Early diagnosis through neonatal screening programs is critical to prevent irreversible outcomes [2].

2-Methylbutyryl-CoA dehydrogenase deficiency (2-MBDD) is a rare organic acidemia caused by short- or branched-chain acyl-CoA dehydrogenase deficiency (SBCAD) in isoleucine metabolic pathway, leading to the accumulation of 2-methylbutyrylcarnitine (C5) in the blood and 2-methylbutyrylglycine in the urine [3, 4]. Most affected individuals are asymptomatic during infancy, but untreated cases may present later with seizures, developmental delay, or cognitive impairments [5, 6]. Metabolic screening started in 2017 at the Growth and Development Research Center (GDRC) affiliated with Tehran University of Medical Sciences [7]. Due to screening in a limited area, there are definitely no comprehensive statistics of metabolic patients in Iran, but according to the recorded information of the screened cases from 2017 to 2022, 102,449 newborns were screened in this center, and no cases of 2-MBDD were identified or registered. This report presents the first documented case of 2-MBDD in Iran, identified through the national neonatal screening program. It underscores the significance of early detection and timely intervention in improving clinical outcomes for rare metabolic disorders.

## Materials and methods

### Clinical features

A 4-day-old male neonate was identified through the neonatal screening program conducted at the Growth and Development Research Center. He was delivered by cesarean section at term, with a birth weight of 2.460 kg, height of 47 cm, normal head circumference and Apgar scores. His mother had a history of hypothyroidism, and he was the first child of healthy, non-consanguineous Iranian parents. The infant was asymptomatic at birth.

Initial metabolic screening was performed on dried blood spots (DBS) using electrospray ionization tandem mass spectrometry (ESI-MS/MS) on day 4 of life. DBS samples were collected from each neonate via heel prick on Whatman 903 filter paper cards between 48 and 72 h of life, in accordance with standard newborn screening protocols. The DBS cards were air-dried at room temperature for 3 h and stored at − 20 °C until analysis.

Targeted metabolomic profiling of amino acids and acylcarnitines was performed using a Shimadzu LCMS-8045 triple quadrupole LC/MS system. A validated commercial kit for newborn screening, the MassChrom^®^ Newborn Screening Kit (57000 F, non-derivatized, Chromsystems Instrument and Chemicals, Germany) was employed for analysis. Urine organic acid analysis was conducted via gas chromatography-mass spectrometry (GC/MS). Organic acids were extracted and derivatized as trimethylsilyl (TMS) esters prior to analysis following standard procedures [8].

Quality control for both ESI-MS/MS and GC-MS analyses was ensured through regular calibration of instruments using standard reference materials and internal controls, according to the laboratory’s standard operating procedures. All assays met the quality assurance standards of the Growth and Development Research Center.

Comprehensive clinical evaluations included fundoscopy, hearing tests, electroencephalography (EEG), echocardiography, and brain magnetic resonance imaging (MRI).

### DNA extraction and Whole-exome sequencing (WES)

After obtaining informed consent, genomic DNA was extracted from peripheral blood using standard protocols. Whole-exome sequencing (WES) was performed on an Illumina HiSeq 4000 platform, with an average read depth of 150X. Library preparation used the Agilent SureSelectXT Human All Exon V6 kit (Agilent Technologies, Santa Clara, CA, USA), and sequencing was carried out on an Illumina HiSeq 2500 platform (Illumina, San Diego, CA, USA) with 100 bp paired-end reads. The mean coverage across exonic regions exceeded 100X, ensuring high-quality sequencing data suitable for downstream analyses.

Sequencing data were aligned to the human reference genome (GRCh37/hg19) using the Burrows-Wheeler Aligner (BWA, version 0.7.17). Variant calling was performed using the Genome Analysis Toolkit (GATK, version 4.1.7.0) to identify single nucleotide variants (SNVs) and small insertions/deletions (indels). Variants were annotated using ANNOVAR (version 2019Sep25), and filtered based on a minor allele frequency (MAF) < 1% in population databases, including (1000 Genomes, ExAC, gnomAD). Alignment and variant calling were conducted using the GRCh38 reference genome. Variants with a MAF > 0.01 were filtered out using population frequency data from the Exome Aggregation Database (gnomAD; https://gnomad.broadinstitute.org/), Exome Aggregation Consortium (ExAC; http://exac.broadinstitute.org*)*, and Exome Sequencing Project 6500 (ESP6500; http://evs.gs.washington.edu/EVS/*).* Variant classification followed the American College of Medical Genetics and Genomics (ACMG) 2015 guidelines.

To assess variant pathogenicity, multiple in silico prediction tools were used, including MutationTaster (http://www.mutationtaster.org/*)*, SIFT (https://sift.bii.a-star.edu.sg/*)*, PROVEAN (http://provean.jcvi.org/index.php*)*, and CADD (https://cadd.gs.washington.edu/home). Candidate variants were further validated by Sanger sequencing, with segregation analysis performed to confirm inheritance patterns within affected families.

## Results

Initial ESI-MS/MS analysis revealed an elevated 2-methylbutyrylcarnitine (C5) level of 0.71 µmol/L (reference range: 0.53–0.68 µmol/L), which increased to 1.013 µmol/L upon repeat testing. Gas chromatography-mass spectrometry (GC-MS) confirmed the presence of 2-methylbutyrylglycine in urine, consistent with 2-methylbutyryl-CoA dehydrogenase deficiency (2-MBDD). No significant excretion of 2-ethylhydracrylic acid was detected. Amino acid profiling showed normal results (Table 1).

**Table 1:** Biochemical findings in a neonate with 2-methylbutyryl-CoA dehydrogenase deficiency (2-MBDD)

Parameter (sample/test type)	Result	Reference range (µmol/L or as indicated)
2-Methylbutyrylcarnitine (DBS, NBS)	0.71	0.53–0.68
2-Methylbutyrylcarnitine (DBS, Follow-up)	1.013	0.39–0.57
C5/C2	0.13	> 0.05
C5/C3	0.88	> 0.64
2-Methylbutyrylglycine (Urine, Follow-up)	18.52	0.2 (mmol/mol creatinine)
2-Ethylhydracrylic acid (Urine, Follow-up)	Not detected	Not detected
Amino acids (Plasma, Follow-up)	Normal	Normal

Abbreviations: NBS, newborn screening; DBS, dried blood spot

The patient was initiated on a low-isoleucine diet, restricting dietary isoleucine to 50 mg/kg/day, using an isoleucine-free formula supplemented with 100 mg/kg/day of L-carnitine, with continued breastfeeding and regular nutritional monitoring to ensure adequate caloric intake and growth until 18 months of age. Due to the absence of clinical symptoms and stable biochemical parameters, the patient was gradually transitioned to a standard diet thereafter, with ongoing monitoring.

Clinical follow-ups every three months demonstrated normal growth, and neurodevelopmental progress, with age-appropriate motor and communication milestones. Serial biochemical assessments—including liver, kidney, and thyroid function tests, urinalysis, and electrolyte panels—remained within normal limits. Ophthalmologic, audiological, EEG, echocardiography, and brain MRI evaluations revealed no abnormalities. Following therapeutic intervention with a low-isoleucine diet and carnitine supplementation, the patient’s C5 level stabilized at 0.4 µmol/L, a value between normal and pathological thresholds.

**Fig. 1:**
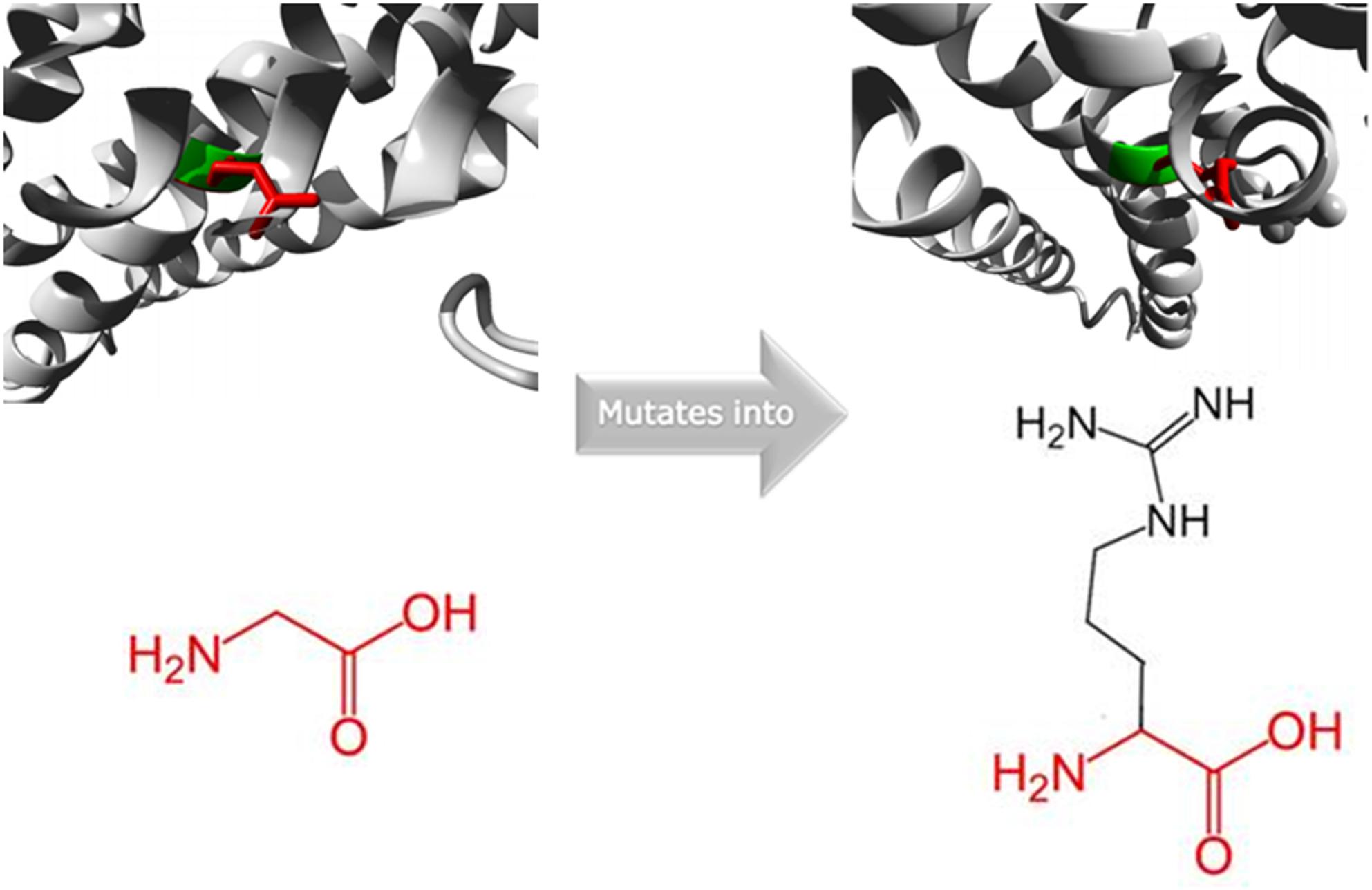
Structural model of the ACADSB protein showing the wild-type glycine (green) and mutant arginine (red) residues at position 303, highlighting altered charge distribution

WES identified a homozygous missense variant in the *ACADSB* gene (c.907G > C; p.G303R). This variant results in the substitution of glycine with arginine (p.Gly303Arg). Based on ACMG guidelines, this variant was classified as likely pathogenic (LP) due to PM1 (located in a mutational hot spot), PM2 (absent from population databases or present at very low frequency), PM5 (Novel missense change at an amino acid residue where a different missense change) and PP3 (predicted deleterious by multiple in silico tools). This missense change is predicted to alter the local charge distribution and hydrophobicity of the protein, potentially impairing its folding stability and catalytic function (Fig. 1).

## Discussion

This study reports the first documented case of 2-methylbutyryl-CoA dehydrogenase deficiency (2-MBDD) in Iran, caused by a novel homozygous missense variant (c.907G >C; p.G303R) in the *ACADSB* gene. The substitution of glycine with arginine at position 303 likely disrupts enzyme function by altering protein stability and charge distribution, as predicted by in silico tools including CADD (score: 28.6), SIFT, and Mutation Taster [4, 8]. Compared to known variants, such as c.443 C >T (p.T148I) reported in East Asian populations [9], and c.1159G >A (E387K) reported by Alfardan et al. [5], p.G303R occurs in a conserved functional domain, suggesting a distinct structural impact that may influence substrate binding or catalytic efficiency. Data from ClinVar indicate that p.G303R is absent from global populations (PM2), reinforcing its novelty and potential pathogenicity [10].

The absence of clinical symptoms in the neonatal period highlights the critical role of newborn screening in early identification of such disorders [11]. Early intervention with a low-isoleucine diet and carnitine supplementation prevented neurological and metabolic complications [12], as observed in our patient’s normal developmental milestones. As previously reported in a neonate identified through newborn screening [13], early detection of 2-MBDD allows for timely intervention and prevents long-term complications. In Iran, where consanguineous marriages account for approximately 38% of unions [14], population-specific genetic studies are essential to identify rare variants. Interestingly, the proband’s parents were non-consanguineous, an uncommon finding for such a rare condition. This raises the possibility that the novel homozygous missense variant (c.907G >C; p.G303R) in the *ACADSB* gene may result from a founder effect or represent a mutational hotspot, warranting further investigation in the Iranian population.

Our findings highlight the feasibility and importance of expanding national newborn screening programs in Iran and other resource-limited settings. Integrating MS/MS-based screening for rare metabolic disorders can be achieved within current infrastructures with appropriate training and resource allocation. Given the high rate of consanguinity in Iran, developing regional variant databases such as Iranome would improve diagnostic precision and enable more effective genetic counseling [15].

This study’s integration of genetic and metabolic screening within a national program demonstrates the feasibility of detecting rare metabolic disorders in resource-limited settings. The identification of a novel *ACADSB* variant enriches the global repository of disease-associated mutations and contributes to the molecular understanding of 2-MBDD. Since the Middle East remains underrepresented in global genetic databases, studies such as ours provide essential population-specific data to inform both personalized medicine and public health strategies [16].

The main limitation of this study is its single-case nature, which restricts generalizability. Functional validation of the p.G303R variant through enzyme activity assays or structural modeling was not feasible due to limited access to specialized biochemical testing. Nonetheless, the variant meets several ACMG/AMP criteria for likely pathogenicity, including its absence from population databases (PM2), localization in a conserved functional domain (PM1), missense change at a residue where another pathogenic variant has been reported (PM5), and multiple deleterious in silico predictions (PP3). Moreover, the biochemical profile—characterized by elevated 2-methylbutyrylcarnitine (C5) on MS/MS and 2-methylbutyrylglycine on GC-MS—provides strong supportive evidence for its clinical relevance. Long-term follow-up is warranted, as some 2-MBDD cases may develop symptoms later in life.

Previous studies in Iran have identified mutations in genes such as *CYP21A2*, *GJB2*, and *TMC1* [17–19], suggesting that c.907G >C may reflect a founder effect or mutational hotspot in this population. Future research involving larger cohorts and functional assays will help clarify its prevalence, biochemical impact, and clinical implications.

This study emphasizes the critical role of newborn screening and genetic testing in the early diagnosis and management of inherited metabolic disorders. Early detection allows for timely therapeutic intervention and prevention of serious complications. The identification of a novel *ACADSB* gene variant not only enriches the understanding of 2-MBDD but also highlights the need for broader genetic studies and database development in Iran. Expanding neonatal screening coverage and integrating molecular analyses will be key steps toward improving diagnostic accuracy and patient outcomes [20].

## Data Availability

The datasets generated and analyzed during this study (including metabolic screening results, whole-exome sequencing data, and clinical evaluations) are not publicly available due to patient confidentiality and ethical restrictions. However, anonymized data may be available from the corresponding author (Saeideh Abdolahpour, abdolahpours@yahoo.com) upon reasonable request, subject to approval by the Ethics Committee of Tehran University of Medical Sciences.

## Abbreviations

2MBCDD: 2-Methylbutyryl-CoA Dehydrogenase Deficiency
ACADSB: Acyl-CoA Dehydrogenase Short/Branched Chain
ACMG: American College of Medical Genetics and Genomics
BWA: Burrows-Wheeler Aligner
C5: 2-Methylbutyrylcarnitine
CADD: Combined Annotation Dependent Depletion
DBS: Dried Blood Spots
EEG: Electroencephalography
ESI-MS/MS: Electrospray Ionization Tandem Mass Spectrometry
ExAC: Exome Aggregation Consortium
GATK: Genome Analysis Toolkit
GC/MS: Gas Chromatography-Mass Spectrometry
GDRC: Growth and Development Research Center
gnomAD: Genome Aggregation Database
IEMs: Inborn Errors of Metabolism
LP: Likely Pathogenic
MAF: Minor Allele Frequency
MRI: Magnetic Resonance Imaging
SBCAD: Short/Branched-Chain Acyl-CoA Dehydrogenase
SIFT: Sorting Intolerant From Tolerant
SNVs: Single Nucleotide Variants
WES: Whole-Exome Sequencing
